# Expression of one important chaperone protein, heat shock protein 27, in neurodegenerative diseases

**DOI:** 10.1186/s13195-014-0078-x

**Published:** 2014-12-17

**Authors:** Xuekai Zhang, Jing Shi, Jinzhou Tian, Andrew C Robinson, Yvonne S Davidson, David M Mann

**Affiliations:** The Third Department of Neurology, Dongzhimen Hospital, Beijing University of Chinese Medicine, No. 5 Haiyuncang Street, Dongcheng District, Beijing, 100700 China; Clinical and Cognitive Sciences Research Group, Institute of Brain, Behaviour and Mental Health, Faculty of Medical and Human Sciences, University of Manchester, Salford Royal Hospital, Salford, M6 8HD UK; Beijing University of Chinese Medicine Neurology Centre, Dongzhimen Hospital, Beijing University of Chinese Medicine, 5 Haiyuncang Street, Beijing, 100700 People’s Republic of China China; Clinical and Cognitive Neuroscience Research Group, University of Manchester, Salford Royal Foundation NHS Trust, Salford, M6 8HD UK

## Abstract

**Introduction:**

Many neurodegenerative diseases are characterised by accumulations of misfolded proteins that can colocalise with chaperone proteins (for example, heat shock protein 27 (HSP27)), which might act as modulators of protein aggregation.

**Methods:**

The role of HSP27 in the pathogenesis of neurodegenerative disorders such as frontotemporal lobar degeneration (FTLD), Alzheimer’s disease (AD) and motor neuron disease (MND) was investigated. We used immunohistochemical and Western blot analysis to determine the distribution and amount of this protein in the frontal and temporal cortices of diseased and control subjects.

**Results:**

HSP27 immunostaining presented as accumulations of granules within neuronal and glial cell perikarya. Patients with AD and FTLD were affected more often, and showed greater immunostaining for HSP27, than patients with MND and controls. In FTLD, there was no association between HSP27 and histological type. The neuropathological changes of FTLD, AD and MND were not immunoreactive to HSP27. Western blot analysis revealed higher HSP27 expression in FTLD than in controls, but without qualitative differences in banding patterns.

**Conclusions:**

The pattern of HSP27 immunostaining observed may reflect the extent of ongoing neurodegeneration in affected brain areas and is not specific to FTLD, AD or MND. It may represent an accumulation of misfolded, damaged or unwanted proteins, awaiting or undergoing degradation.

## Introduction

Many neurodegenerative diseases, also termed as *protein conformational diseases* [1], are characterised by accumulations of misfolded proteins that often share morphological and biochemical features and can colocalise with several other proteins, including various chaperone proteins. The accumulation of misfolded proteins may adversely affect neuronal connectivity and plasticity and trigger cell death signalling pathways [2]. These misfolded proteins include amyloid-β protein (Aβ) and tau in Alzheimer’s disease (AD); α-synuclein and synphilin 1 in Parkinson’s disease (PD); polyglutamine (polyQ)-expanded huntingtin in Huntington’s disease; transactive response DNA binding protein 43 (TDP-43) and copper-zinc superoxide dismutase 1 in motor neuron disease (MND); and tau, TDP-43 and fused in sarcoma in frontotemporal lobar degeneration (FTLD). These aggregates may consist of oligomeric complexes of non-native secondary structures and demonstrate poor solubility in aqueous or detergent solvents [3]. Chaperone proteins, such as heat shock proteins (HSPs), play a prime role in protein homeostasis by binding to substrates at risk, thereby keeping them in a state competent for either refolding or degradation [4]. Chaperone proteins have therefore been implicated as potent modulators of protein conformational disorders, suppressing toxicity of misfolding proteins and modifying early events in the aggregation process in a cooperative and sequential manner reminiscent of their functions in *de novo* protein folding [5,6].

HSP27, also known as HSPB1, is one of the best studied members of the small HSP family. It functions as a molecular chaperone, aiding the refolding of non-native proteins, and plays a critical role in stabilisation of the cytoskeleton through interactions with several cytoskeletal components, such as actin, intermediate filaments and microtubules [7]. Normally, when functioning as a molecular chaperone, HSP27 can bind non-native substrates in an ATP-independent manner, which is quite different from most HSPs [8]. However, for substrate release, it requires the help of ATP-dependent chaperone proteins, such as HSP70 and HSP104 [9]. Additionally, HSP27 can exhibit a number of cytoprotective properties in cell culture, attenuating apoptosis through interaction with proapoptotic proteins such as Daxx, cytosolic cytochrome *c* and caspase 3, as well as suppressing aggregate formation [10,11].

HSP27 has been implicated in various neurodegenerative diseases. For example, a highly induced expression of HSP27 has been reported in the brains of aged persons and of patients with AD, being present in proliferating astrocytes, especially in AD in those areas rich in senile (Aβ) plaques. Neurofibrillary tangles, Hirano bodies and some hippocampal neurons have also been reported to be HSP27-immunoreactive. However, in control brains, HSP27 immunoreactivity was restricted to blood vessels and to occasional astrocytes in the white matter. Similarly, patients with other types of dementia (Parkinson–dementia complex, multi-infarct dementia and normal pressure hydrocephalus) showed less expression of HSP27 in reactive astrocytes than that in AD, but more than that in controls. Occasional immunoreactive HSP27 astrocytes have also been reported to be present in Parkinson disease [12].

Although the physiological relationship between HSP27 and misfolded proteins in neurodegenerative diseases has been studied intensively *in vivo* and *in vitro*, any potential role of HSP27 in the pathogenesis of neurodegenerative diseases, and especially FTLD, is still incompletely understood. The principal aim of this study was to investigate the possible role of HSP27 in the pathogenesis of FTLD. This was achieved by comparing the distribution and amount of HSP27 in FTLD, AD, MND and control groups by immunohistochemistry, and by characterising biochemical changes using Western blotting.

## Methods

### Patients

The study group comprised 175 patients, composed of 72 patients with FTLD, 46 with AD, 25 with MND and 32 controls (see Table 1 for demographic characteristics). Patients with FTLD fulfilled the Lund-Manchester criteria [13] for sporadic or familial FTLD and their characteristics were consistent with recent consensus criteria [14]. All had been longitudinally assessed within the Cerebral Function Unit, Salford Royal Hospital, with the Manchester Neuropsychological Test Battery. Data collected on each patient included sex, age at diagnosis and at death, and clinical diagnosis. The clinical diagnosis was confirmed at autopsy [15]. The FTLD patients were histologically classified according to the harmonised classification system for FTLD-TDP [16] into FTLD-TDP type A, FTLD-TDP type B and FTLD-TDP type C (Table 1). The diagnosis of clinical MND (in patients with or without FTLD) was in keeping with El Escorial criteria [17]. Diagnosis of possible or probable AD was made in accordance with the National Institute of Neurological and Communicative Disorders and Stroke–Alzheimer’s Disease and Related Disorders Association criteria [18] and was confirmed pathologically according to Consortium to Establish a Registry for Alzheimer’s disease criteria [19]. Control subjects were obtained from a longitudinal ageing cohort of elderly people without clinical symptoms of dementia. All brains were drawn from the Manchester Brain Bank and had been obtained with full ethical permission of the Manchester Brain Bank Management Committee under Tissue Bank Ethical Agreement 08 conferred by Newcastle and North Tyneside 1 Research Ethics Committee following appropriate consenting procedures by the next of kin.

**Table 1:** **Demographic details** ^**a**^

**Group (** ***n*** **)**	**M/F,** ***n*** **(missing data)**	**Age at death, yr (mean ± SD)**	**Sections available,** ***n***		
			**Frontal**	**Temporal**	**Hippocampal**
Controls (32)	14/18	72.0 ± 21.0	19	32	31
AD (46)	23/23	72.0 ± 6.0	46	41	41
MND (25)	20/5	64.3 ± 12.0	17	25	24
FTLD (72)	41/31	65.6 ± 9.1	71	66	60
FTLD-tau (31)	14/14 (3)	67.2 ± 9.6	31	25	25
*MAPT* (5)	2/3	62.2 ± 5.4	5	4	4
Pick (26)	12/11 (3)	68.3 ± 10.1	26	21	21
FTLD-TDP (41)	26/15	64.7 ± 8.7	40	41	35
Type A (17)	8/9	68.7 ± 4.2	17	17	14
Type B (14)	12/2	58.3 ± 9.0	13	14	12
Type C (10)	6/4	66.5 ± 9.4	10	10	9

^a^AD, Alzheimer’s disease; FTLD, Frontotemporal lobar degeneration; FTLD-*MAPT*, Frontotemporal lobar degeneration with mutations in the microtubule-associated protein tau; MND, Motor neuron disease; FTLD-tau Pick, Frontotemporal lobar degeneration tau with Pick bodies; TDP, Transactive response DNA binding protein 43 proteinopathy (subtypes A, B and C according to Mackenzie et al. [16]).

### Immunohistochemistry

Formalin-fixed, paraffin-embedded sections (6 μm) of frontal cortex (Brodmann areas 8/9 (BA8/9)) and/or temporal cortex (BA21/22) with hippocampus (where available; see Table 1) were immunostained for HSP27 using a commercially available rabbit polyclonal HSP27 antibody (ab5579; Abcam, Cambridge, UK). A standard immunostaining procedure was followed [20,21]. Briefly, all deparaffinised sections underwent antigen retrieval in 0.1 M citrate buffer, pH 6.0, by microwave heating for 10 minutes. To quench endogenous peroxidase activity, slides were incubated in 0.3% hydrogen peroxide in methanol for 30 minutes. Sections were then incubated overnight (at 4°C) in a diluted blocking serum (normal goat serum) to mask nonspecific binding sites. This was followed by 1-hour immunostaining with primary polyclonal HSP27 antibody diluted to 1:500. Following three 5-minute washes in phosphate-buffered saline, incubations in biotinylated secondary antibody and avidin–biotin complex were performed for 30 minutes each using a VECTASTAIN ABC kit (Vector Laboratories, Peterborough, UK), The immunoreaction was visualised using 3,3′-diaminobenzidine (Sigma, Poole, Dorset, UK). Sections were lightly counterstained with haematoxylin, dehydrated sequentially and mounted with coverslips using DePeX mounting medium. Further sections were immunostained for LAMP1 (rabbit polyclonal antibody AP1823a; Abgent, San Diego, CA, USA), LCA3 (rabbit polyclonal antibody L8793; Sigma, St Louis, MO, USA), p62 (p62-lck ligand, rabbit polyclonal antibody; BD Biosciences, Oxford, UK) and ubiquitin (rabbit polyclonal antibody Z0458; Dako Cytomation, Ely, UK), employing antibodies at dilutions of 1:200, 1:200, 1:100 and 1:750, respectively.

### Pathological assessment

The degree and distribution of HSP27 immunostaining in both white matter and grey matter were scored semiquantitatively in temporal cortex and hippocampus, and/or frontal cortex, on a 5-point scale (0 = absent; 1 = rare; 2 = mild; 3 = moderate; 4 = severe) using a Leica DMR microscope (×200 magnification, DC 300 F, 10447115; Leica Microsystems, Milton Keynes, UK). The examiner (XK) was blinded to clinical and pathological diagnosis, as well as to the results of the initial scoring of sections, when reexamining the series for a second and third time. Intraclass correlation coefficient and interrater reliability (Cohen’s κ) testing were used to analyse the consistency of scoring over the three assessments. The results showed that there was substantial agreement for ratings performed at each time and for each area (Tables 2 and 3).

**Table 2:** **Analysis of assessment scores by intraclass correlation coefficients** ^**a**^

	**1st vs 2nd assessments**	**1st vs 3rd assessments**	**2nd vs 3rd assessments**	**1st vs 2nd vs 3rd assessments**
Neurons				
ICC	**0.874**	**0.815**	**0.854**	**0.892**
*P*-value	0.000	0.000	0.000	0.000
Grey matter glial cells				
ICC	**0.930**	**0.933**	**0.935**	**0.954**
*P*-value	0.000	0.000	0.000	0.000
White matter glial cells				
ICC	**0.958**	**0.928**	**0.935**	**0.960**
*P*-value	0.000	0.000	0.000	0.000

^a^ICC, Intraclass correlation coefficient. Results with perfect agreement (>0.8) are in boldface.

**Table 3:** **Analysis of assessment scores by interrater reliability**

	**1st vs 2nd assessments**	**1st vs 3rd assessments**	**2nd vs 3rd assessments**
Neurons			
Cohen’s κ	**0.629**	0.598	**0.643**
*P*-value	0.000	0.000	0.000
Grey matter glial cells			
Cohen’s κ	0.444	**0.671**	**0.666**
*P*-value	0.000	0.000	0.000
White matter glial cells			
Cohen’s κ	**0.645**	0.554	**0.625**
*P*-value	0.000	0.000	0.000

Results with substantial agreement (0.61 to 0.80) are in boldface.

### Statistical analysis

All data were analysed using appropriate parametric and nonparametric statistical tests with the SPSS for Windows statistical software package (release 16.0; IBM SPSS, Chicago, IL, USA). In immunohistochemical analysis, the frequency of immunopositive or immunonegative cases in different brain regions between different groups was assessed using Pearson’s χ^2^ test. The severity of HSP27 immunostaining in different brain regions between different groups was analysed using a nonparametric Kruskal-Wallis test with a *post hoc* Mann-Whitney test. To guard against false-positive results, given the high number of statistical tests employed, only *P*-values ≤0.01 were considered significant.

### Western blot analysis

Brain tissues from five patients were studied (Table 4). Clinically, three patients had been diagnosed with various histological and genetic forms of FTLD. Two cases served as controls, one without any significant pathology and one with mild AD-type changes. Fresh-frozen frontal cortex tissue was used for sequential extraction of proteins with specific buffers. Sequential extracts of proteins are produced after several protein preparation steps, including whole-cell lysate and other fractions—low salt fraction, Triton X-100 fraction, sarkosyl fraction and urea fraction. These fractions represent a series containing a decreasing solubility of (potentially interesting) proteins.

**Table 4:** **Cases assessed in Western blot analysis** ^**a**^

**Case**	**Onset, yr**	**Death, yr**	**Duration, yr**	**Clinical diagnosis**	**Pathological diagnosis**
1	43	45	2	FTD + MND	FTLD-TDP B
2	52	65	13	FTD	FTLD-*MAPT*
3	63	74	11	FTD	FTLD-Pick
4	na	80	na	Normal	None
5	na	82	na	Normal	Mild AD

^a^AD, Alzheimer’s disease; FTD, Frontotemporal dementia; FTLD, Frontotemporal lobar degeneration; FTLD-Pick, Frontotemporal lobar degeneration tau with Pick bodies; FTLD-*MAPT*, Frontotemporal lobar degeneration with mutations in the microtubule-associated protein tau; FTLD-TDP B, Frontotemporal lobar degeneration with transactive response DNA binding protein subtype B; MND, Motor neuron disease; na, Not applicable.

Using an adapted protocol [22], sequential fractions were examined by Western blotting for HSP27. Equal volumes (25 μl) of diluted fractions from different samples and 6-μl molecular weight marker/ladder were condensed by 5% stacking gel and resolved by 10% SDS-PAGE. Electrophoresis was performed at around 120 V for 60 minutes using a PowerPac Basic power supply (Bio-Rad Laboratories, Hercules, CA, USA). Once the blue line produced by loading buffer (2× Laemmli sample buffer) had reached the bottom plastic plate, the electrophoresis was stopped. The proteins on the gel were then transferred to nitrocellulose membranes (Amersham Hybond-ECL; GE Healthcare Life Sciences, Little Chalfont, UK) via a ‘semidry’ method (Trans-Blot SD Semi-Dry Transfer Cell; Bio-Rad Laboratories), which employs a PowerPac HC Power Supply (Bio-Rad Laboratories) running at 15 V for 1 hour. Following transfer, membranes were blocked with Tris-buffered saline containing 3% powered milk for 20 to 60 minutes at room temperature and then probed with rabbit polyclonal HSP27 antibody (ab5579; Abcam), which was diluted to 1:3,000 in 3% bovine serum albumin solution with sodium azide overnight at 4°C. Primary antibodies were detected with horseradish peroxidase–conjugated goat anti-rabbit immunoglobulin G (sc-2004, 1:5,000; Santa Cruz Biotechnology, Santa Cruz, CA, USA), and immunoreactive proteins on the blots were revealed by ECL Plus Western blotting detection reagent ECL Imager system (Thermo Scientific, Rockford, IL USA).

## Results

### Cytological observations

In all cases, HSP27 immunoreactivity was seen in the form of clusters of small or coarse granules within the cytoplasm of neurons and glial cells. This pattern of immunostaining was qualitatively similar in all cases, irrespective of diagnosis, though the severity of changes varied from case to case between diagnostic categories and within each diagnostic category.

**Figure 1:**
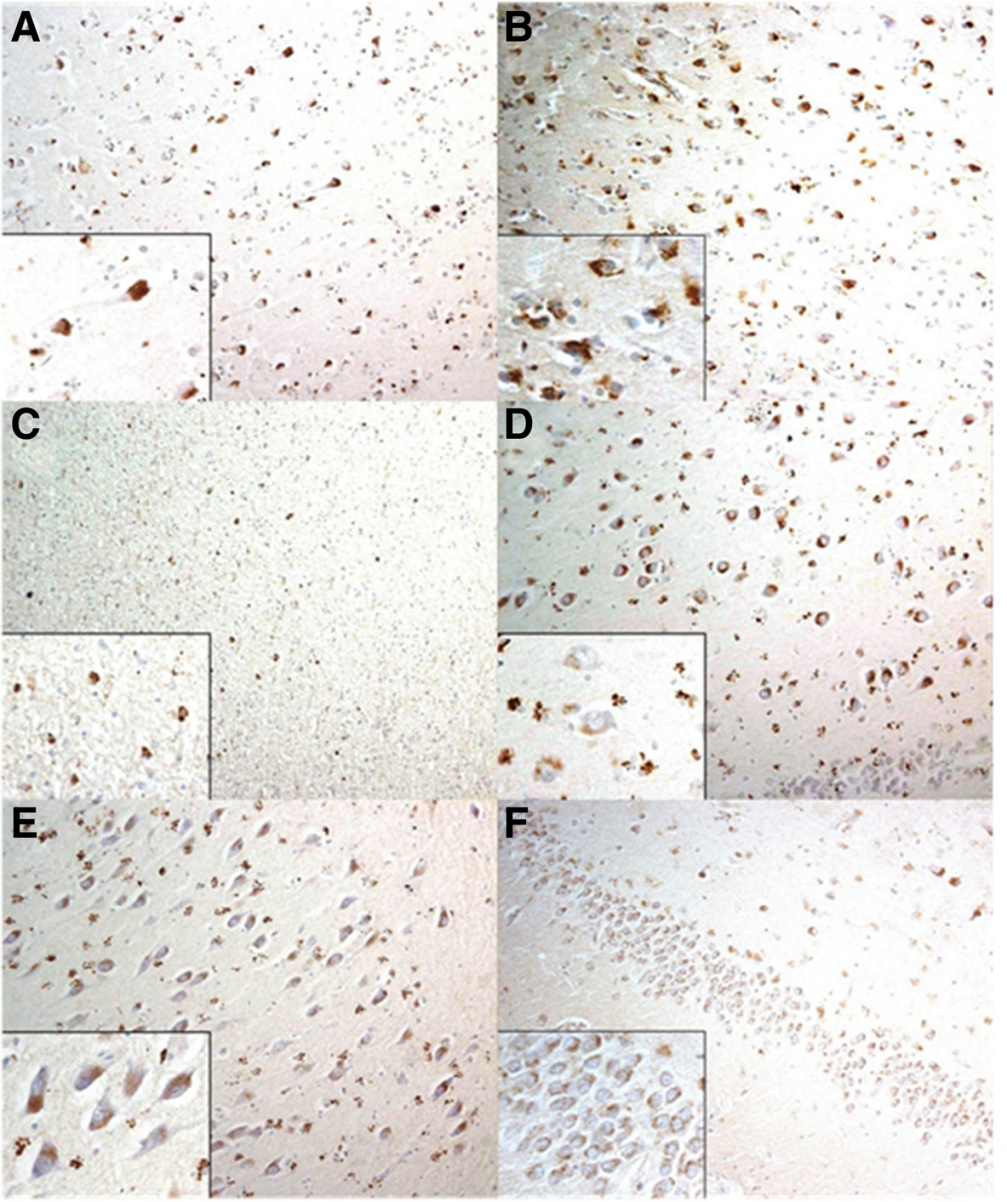
**Characteristic HSP27-positive granules in the cytoplasm of neurons and glial cells in different regions of Alzheimer’s disease cases under light microscopy. (A)** Temporal cortex. **(B)** Frontal cortex. **(C)** White matter of cortex. **(D)** CA4 of hippocampus. **(E)** CA3 of hippocampus. **(F)** Dentate gyrus of hippocampus.

**Figure 2:**
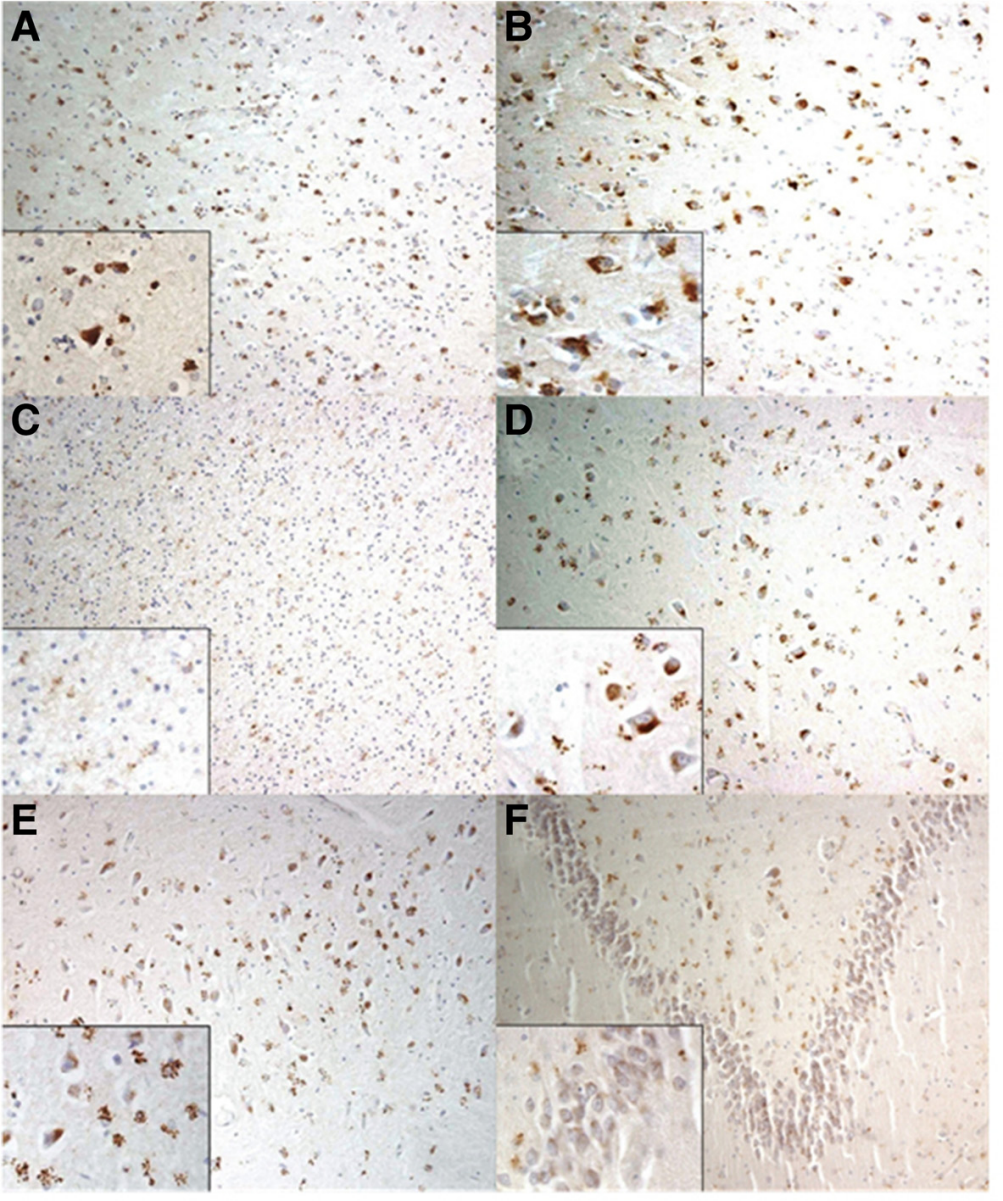
**Characteristic HSP27-positive granules in the cytoplasm of neurons and glial cells of different regions of frontotemporal lobar degeneration cases under light microscopy. (A)** Temporal cortex. **(B)** Frontal cortex. **(C)** White matter of cortex. **(D)** CA4 of hippocampus. **(E)** CA3 of hippocampus. **(F)** Dentate gyrus of hippocampus.

In all patients with FTLD and AD (Figures 1 and 2), there were numerous intensely immunostained, HSP27-positive granules in the cytoplasm of pyramidal neurons of the cerebral cortex, along with abundant diffuse clusters of similar granules within glial cells in both grey and white matter regions. Such changes were particularly severe in cells of the dentate gyrus and the CA4 region of the hippocampus, Similar, though less severe, changes were seen in these same brain areas in patients with MND and in control subjects.

However, there was no HSP 27 immunostaining of senile plaques or neurofibrillary tangles within the frontal and temporal cortices and the hippocampus in patients with AD, nor was there HSP 27 immunostaining of Pick bodies in patients with FTLD-tau, or TDP- 43 immunoreactive neuronal cytoplasmic inclusions in patients with F TLD-TDP, or MND (not shown).

By immunostaining with LAMP1 antibody, we detected granular structures in neurons and glial cells that were similar in appearance to those visualised by HSP27 immunostaining. However, immunostaining with LCA3 antibody failed to reveal any granular structures or staining patterns similar to those detected with HSP27.

### Semiquantitative findings

**Figure 3:**
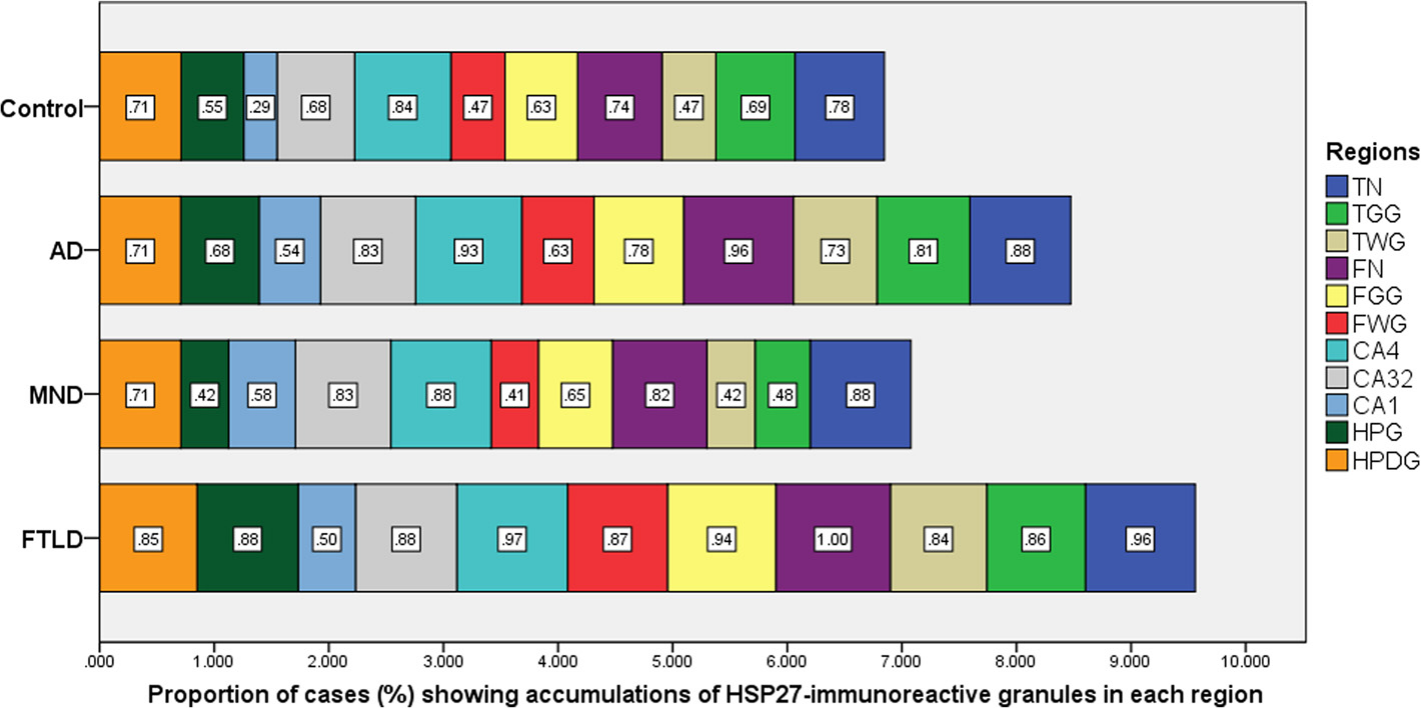
**Proportion of cases with HSP27-positive granules in different brain regions in each group.** AD, Alzheimer’s disease; CA1, neurons of CA1 + subiculum regions of the hippocampus; CA3/2, Neurons of CA3/2 region of the hippocampus; CA4, Neurons of CA4 region of the hippocampus; FGG, Grey matter glial cells of the frontal cortex; FN, Neurons of the frontal cortex; FTLD, Frontotemporal lobar degeneration; FWG, White matter glial cells of the frontal cortex; HPDG, Granular cells of the dentate gyrus of the hippocampus; HPG, Glial cells of the hippocampus; MND, Motor neuron disease; TGG, Grey matter glial cells of the temporal cortex; TN, Neurons of the temporal cortex; TWG, White matter glial cells of the temporal cortex.

The proportion of cases showing HSP27 immunoreactive neurons and/or glial cells in the different brain regions analysed (irrespective of the intensity of staining of individual neurons or glial cells; see below) is shown in Figure 3. The proportion of cases in each diagnostic category showing HSP27 immunoreactive neurons and/or glial cells was highly significantly different between FTLD (overall), AD, MND and control groups for frontal cortex (neurons: χ^2^ = 20.8, *P* =0.001; glial cells of grey matter: χ^2^ = 15.9, *P* =0.003; glial cells of white matter: χ^2^ = 28.8, *P* =0.000) and temporal cortex (glial cells of grey matter: χ^2^ = 15.8, *P* =0.001; glial cells of white matter: χ^2^ = 23.0, *P* =0.000) and marginally significant for temporal cortical neurons (χ^2^ = 6.8, *P* =0.080). However, there were no significant differences between diagnostic groups for the proportion of cases in each diagnostic category showing HSP27 immunoreactive neurons in hippocampus CA4 (χ^2^ = 5.0, *P* =0.170), CA3/2 (χ^2^ = 6.0, *P* =0.113), CA1 and subiculum (χ^2^ = 6.1, *P* =0.106) regions or granule cells of the dentate gyrus (χ^2^ = 4.1, *P* =0.251), though glial cells of hippocampus did show a significant difference between groups (χ^2^ = 21.870, *P* =0.000).

*Post hoc* analysis showed the proportion of cases affected was higher in FTLD subjects than in controls in all regions (temporal cortex: neurons, *P* =0.008; glial cells of grey matter, *P* =0.039; glial cells of white matter, *P* =0.000; frontal cortex: neurons, *P* =0.000; glial cells of grey matter, *P* =0.000; glial cells of white matter: *P* =0.000; hippocampus: glial cells of CA4, *P* =0.000). Similarly, there was a higher proportion of cases affected in FTLD than MND in glial cells of both grey matter (*P* =0.000) and white matter (*P* =0.000) of both the frontal and temporal cortices (*P* =0.000 in every instance) and for glial cells of the hippocampus (*P* =0.000).

There was a significantly higher proportion of cases affected in FTLD than in AD in respect of glial cells of both grey matter (*P* =0.009) and white matter (*P* =0.002) of the frontal cortex, and marginally so for glial cells of the hippocampus (*P* =0.013). There was a higher proportion of cases affected in AD than in controls for the temporal cortex white matter glial cells (*P* =0.012) and the frontal cortex neurons (*P* =0.009). There were no significant differences between MND and control cases in any region, except for neurons in CA1 and subiculum (*P* =0.001).

Subgroup analysis between FTLD-tau and FTLD-TDP groups, between FTLD-*MAPT* and FTLD-Pick groups, and between FTLD-TDP A, B and C groups all showed no significant differences in the frequencies of HSP27-positive cases, except for comparison between FTLD-TDP subtypes A, B and C for glial cells in white matter of the frontal cortex (χ^2^ = 11.9, *P* =0.003), where a higher proportion of FTLD-TDP A cases (*P* =0.005) were affected compared to FTLD-TDP C cases.

**Figure 4:**
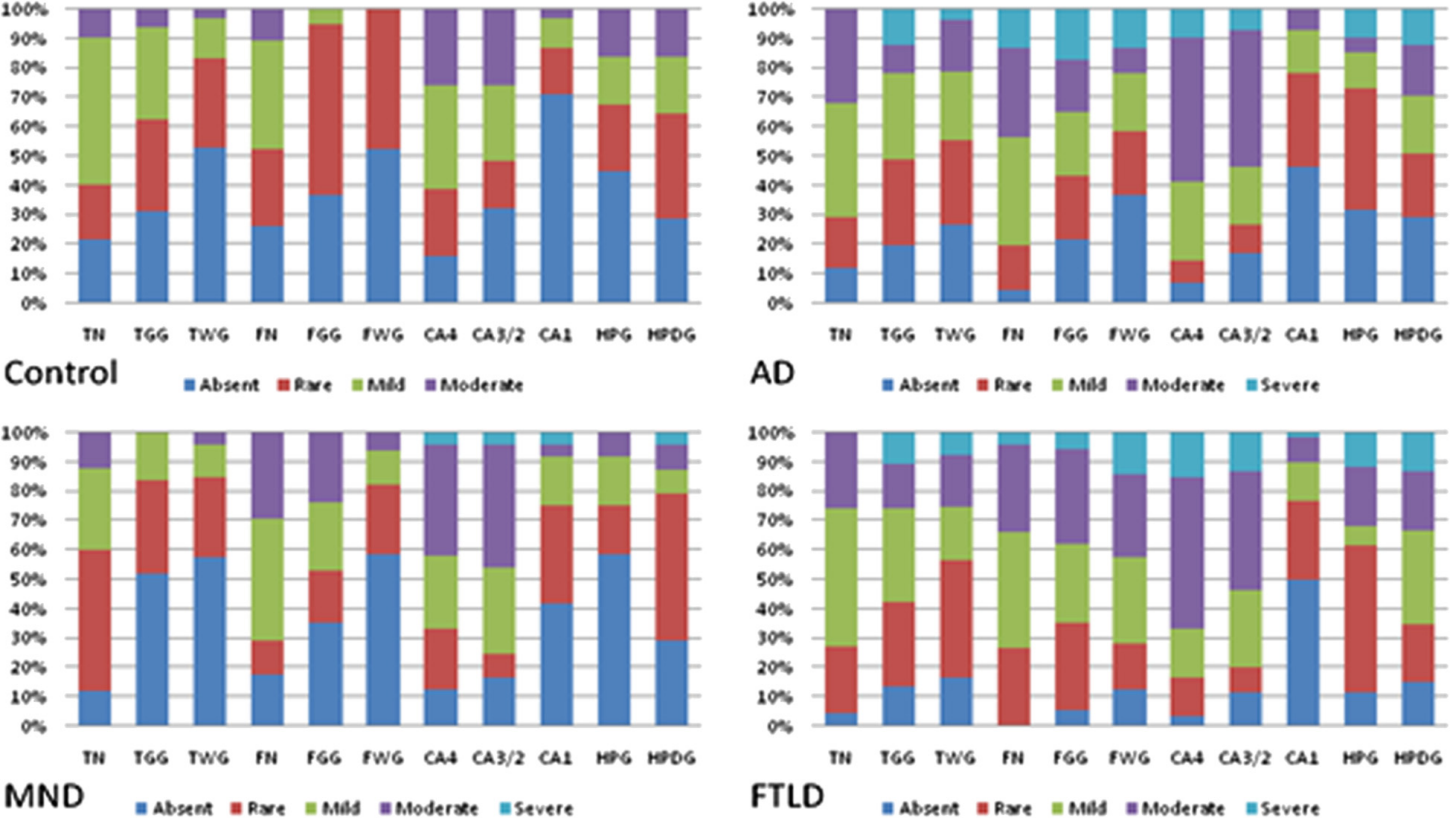
**Severity of different rating scores of HSP27 staining in different brain regions in each group.** AD, Alzheimer’s disease; CA1, Neurons of the CA1 and subiculum regions of the hippocampus; CA3/2, Neurons of the CA3/2 region of the hippocampus; CA4, Neurons of the CA4 region of the hippocampus; FGG, Grey matter glial cells of the frontal cortex; FN, Neurons of the frontal cortex; FTLD, Frontotemporal lobar degeneration; FWG, White matter glial cells of the frontal cortex; HPDG, Granule cells of the dentate gyrus of the hippocampus; HPG, Glial cells of the hippocampus; MND, Motor neuron disease; TGG, Grey matter glial cells of the temporal cortex; TN, Neurons of the temporal cortex; TWG, White matter glial cells of the temporal cortex.

The severity of HSP27 immunoreactivity in neurons and glial cells in the different brain regions in all four diagnostic groups was compared by Kruskal-Wallis test, with *post hoc* Mann-Whitney testing where significant. In general, analysis showed that neurons and glial cells in patients with AD and FTLD were more severely affected than patients with MND Q12 and control subjects (Figure 4). Comparisons of the FTLD (overall), AD, MND and control groups showed significant differences in the severity of HSP27 rating values for neurons (*F*_(3, 162)_ =10.7, *P* =0.013), grey matter glial cells (*F*_(3, 162)_ =21.7, *P* =0.000) and white matter glial cells (*F*_(3, 162)_ =25.8, *P* =0.000) of the temporal cortex, and for neurons (*F*_(3, 151)_ =18.9, *P* =0.001), grey matter glial cells (*F*_(3, 151)_ =29.2, *P* =0.000) and white matter glial cells (*F*_(3, 151)_ =46.0, *P* =0.000) of the frontal cortex. In the hippocampus, there were also significant differences in the severity of HSP27 rating for neurons of CA4 (*F*_(3, 154)_ =17.8, *P* =0.000) region and for glial cells (*F*_(3, 154)_ =14.9, *P* =0.002) and granule cells of dentate gyrus (*F*_(3, 154)_ =11.8, *P* =0.008).

*Post hoc* testing showed that there was significantly greater HSP27 immunostaining of neurons of the temporal cortex in FTLD compared to controls (*P* =0.016) and MND (*P* =0.007) groups, and marginally significantly greater staining in AD compared to MND (*P* =0.028). Similarly, there was significantly greater HSP27 immunostaining of glial cells of grey matter of the temporal cortex in AD compared to controls (*P* =0.002).

In the frontal cortex, there was significantly greater HSP27 staining neurons of the frontal cortex in FTLD (*P* =0.004) and AD groups (*P* =0.001) compared to controls. There was also significantly greater HSP27 staining in glial cells of grey matter in FTLD compared to MND (*P* =0.010), and in glial cells of the white matter in FTLD compared to both MND (*P* =0.000) and AD (*P* =0.002) groups.

In the hippocampus, there was significantly greater HSP27 staining in CA4 region in the FTLD group (*P* =0.000) and the AD group (*P* =0.002) compared to controls, and in the FTLD group compared to the MND group (*P* =0.009). There was also significantly greater HSP27 staining in FTLD compared to AD (*P* =0.030), control (*P* =0.009) and MND (*P* =0.001) for glial cells of the hippocampus, and for granule cells of dentate gyrus in FTLD compared to MND (*P* =0.002) and control (*P* =0.007).

However, subgroup analysis between FTLD-tau and FTLD-TDP groups, between FTLD-*MAPT* and FTLD-Pick groups, and between FTLD-TDP type A, B and C groups, all showed no significant differences in the severity of HSP27 rating values, except for significant differences between FTLD-tau and FTLD-TDP groups for grey matter glial cells of the frontal cortex (*P* =0.006).

### Western blotting

**Figure 5:**
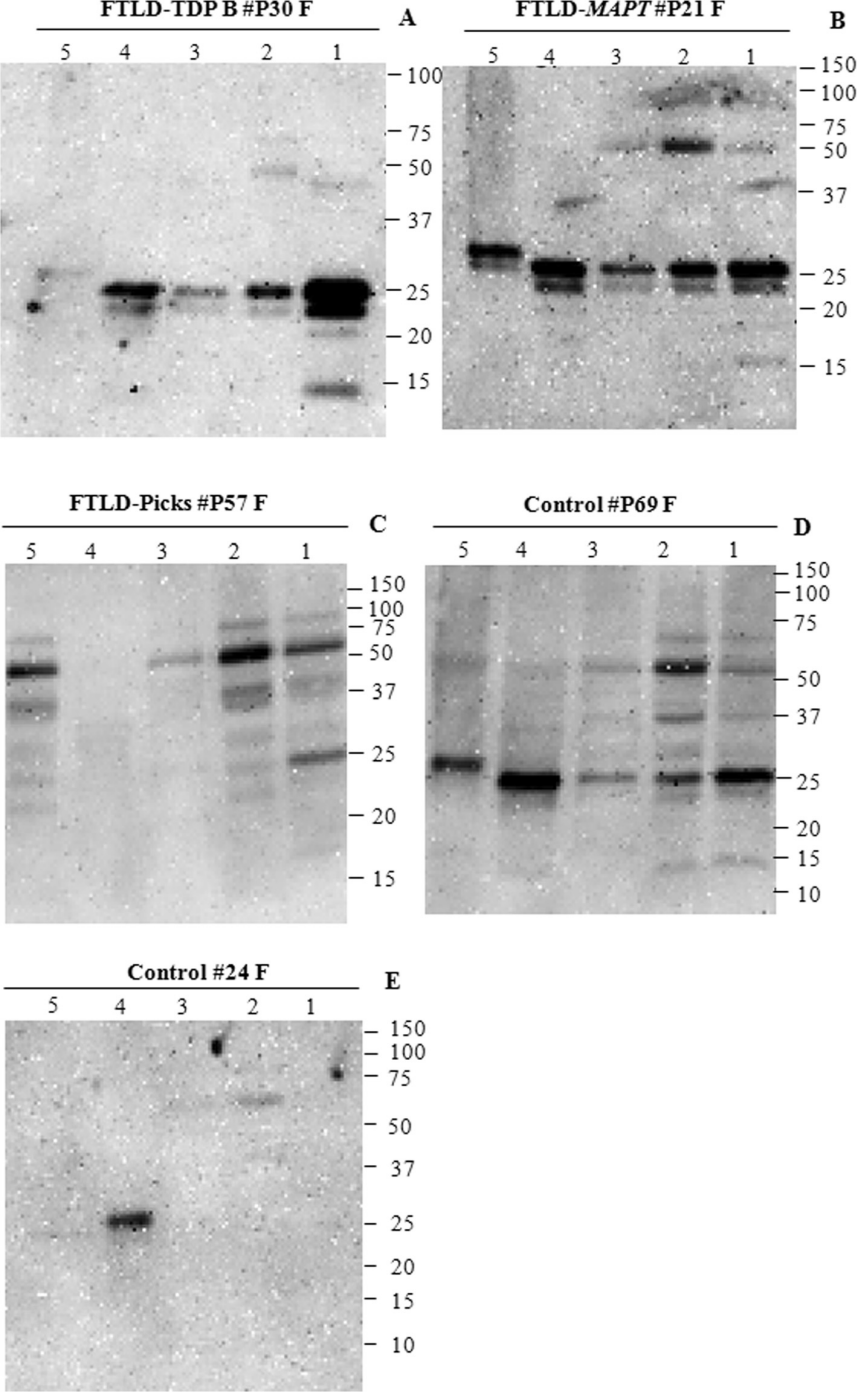
**Immunoblotting of HSP27 in frontal cortex from FTLD and control cases. (A)** Frontotemporal lobar degeneration transactive response DNA binding protein (FTLD-TDP), case 1. **(B)** FTLD-*MAPT* case 2. **(C)** FTLD-Pick case 3. **(D)** Control case 4. **(E)** Control case 5. Lane 1: Whole-cell lysate; lane 2: low salt fraction; lane 3: Triton X-100 fraction; lane 4: sarkosyl fraction; lane 5: urea fraction.

Western blotting of each fraction of the frontal cortex from the two samples showed two normal bands at approximately 50 kDa and approximately 25 kDa, respectively (Figures 5D and 5E). Although there was higher HSP27 expression in FTLD cases than in control cases, this was without any change in banding patterns (Figures 5A, 5B and 5C). Where there were matched samples, there was good correlation between the intensity of staining on Western blots and the severity of HSP27 immunoreactive changes on immunohistochemical stains (Table 5). There were no obvious differences in HSP27 expression between the pathological subtypes in FTLD (Figures 5A, 5B and 5C).

**Table 5:** **Semiquantitative rating of immunoblotting of HSP27 and comparison with immunohistochemistry** ^**a**^

	**Approximately 25-kDa band**					**Approximately 50-kDa band**					**IHC rating scores**			
**Case**	**WL**	**LS**	**TX**	**SARK**	**UF**	**WL**	**LS**	**TX**	**SARK**	**UF**	**F-N**	**F-GG**	**F-WG**	**F-Astro**
1	++++	++	+	++	+/-	+/-	+/-	-	-	-	3	3	2	3
2	+++	+++	++	+++	+++	+	++	+	-	-	3	3	3	3
3	+	+/-	-	-	+/-	++	+++	+	-	++	2	3	4	4
4	+++	++	+	+++	++	+/-	++	+/-	-	+/-	nd	nd	nd	nd
5	-	-	-	++	-	-	+/-	-	-	-	1	1	1	2

^a^+/++/+++ of bands; +/- very faint bands; - no bands at all; F-Astro, Astrocytes of frontal cortex; F-GG, Grey matter glial cells of frontal cortex; F-N, Neurons of frontal cortex; F-WG, White matter glial cells of frontal cortex; IHC, Immunohistochemistry; LS, Low salt fraction; nd, not done; SARK, Sarkosyl fraction; TX, Triton X-100 fraction; UF, Urea fraction; WL, Whole protein fractions.

## Discussion

Although the physical and physiological relationships between chaperone proteins, such as HSP27, and misfolded proteins has been studied intensively, a role for HSP27 in the pathogenesis of neurodegenerative disease is still incompletely understood. In the present study, the expression of HSP27 in FTLD, AD, MND and normal controls was analysed through immunohistochemical staining with HSP27 antibody and Western blotting. HSP27 is usually induced in response to cellular stress, but it can also stabilise the actin cytoskeleton, attenuate apoptosis, participate in cell differentiation and survival when phosphorylated [23], and promote cell cycle reentry into the S-phase by facilitating ubiquitination and degradation of the cell cycle inhibitor p27^Kip1^ [24]. The affinity of HSP27 for proteins to be chaperoned is modulated by its phosphorylation and oligomerisation status [25].

Our present results show that the expression patterns of HSP27 in these neurodegenerative diseases are quite different. Patients with FTLD and AD showed numerous intensely immunostained, HSP27-positive granules in the cytoplasm of pyramidal neurons of the cerebral cortex, along with diffuse clusters of similar granules within glial cells. Such changes were particularly severe in the CA4 region of the hippocampus and in cells of the dentate gyrus. Subgroup analysis showed that there were no differences in the patterns of immunostaining between FTLD-tau and FTLD-TDP cases. However, in cases of MND and normal controls, we found less extensive accumulation of HSP27 immunoreactive granules in these same brain areas. These observations probably reflect the greater ongoing neurodegeneration in these brain areas in AD and FTLD compared to MND.

There have been few previous studies in which investigators have evaluated HSP27 reactivity in neurodegenerative disease, and then only in relationship to AD. To our knowledge, there have been no such studies in FTLD. In general, these studies have shown there to be an increase in expression of HSP27 in AD brain tissue or in immunolabelling of pathological structures [12,25-27]. Using a rabbit anti-HSP27 antiserum raised against a hybrid protein containing the N-terminal part of the murine HSP27 (amino acids 1 to 110) and the C-terminal part of the human HSP27 (amino acids 111 to 208), Renkawek and co-workers showed a highly induced expression of HSP27 in many proliferating astrocytes in affected regions of cerebral cortex, especially in those areas rich in senile plaques [12]. They also reported neurofibrillary tangles, Hirano bodies and some hippocampal neurons to be immunopositive [12]. The expression of HSP27 correlated with the severity of AD-specific morphological changes and the duration of dementia [12]. Shimura and co-workers partially confirmed these findings, demonstrating a coimmunoprecipitation of HSP27 with hyperphosphorylated tau protein from AD brain, but not with nonphosphorylated tau from normal brain homogenates [25]. However, in another study, a diffuse, rather than granular, HSP27 immunostaining was observed in a subpopulation of astrocytes and microglial cells associated with senile plaques and cerebral amyloid angiopathy, but this was not seen in neurofibrillary tangles in either the neocortex or hippocampus [27]. In a biochemical study, Bjorkdahl and colleagues reported the expression of HSP27 in AD brain to be increased (by approximately 20%) in homogenates from the medial temporal cortex [26]. Moreover, this increase in HSP27 correlated significantly with the expression levels of the majority of the phosphorylated tau epitopes, but not with AT270, Alz-50 or AT100 [26].

In the present study, we observed numerous intensely immunostained, HSP27-positive granules in the cytoplasm of pyramidal neurons of the cerebral cortex and as diffuse clusters of similar appearing granules within glial cells, in both FTLD and AD. However, in AD, there was no HSP27 immunostaining of senile plaques or neurofibrillary tangles within the frontal and temporal cortices or within the hippocampus. The failure to confirm previous findings [12] (but see [27]) may be partly due to the different HSP27 antibodies used. In the present study, the anti-HSP27 antibody used (ab5579; Abcam) is a rabbit polyclonal raised against a synthetic peptide, LLRGPSWDPFRC, corresponding to amino acids 10 to 21 of human HSP27. It is much more specific than the rabbit anti-HSP27 antiserum raised against a hybrid protein containing the N-terminal part of the murine Hsp25 (amino acids 1 to 110) and the C-terminal part of the human HSP27 (amino acids 111 to 208) used by Renkawek et *al*. [12]. Hence, the staining of plaques (amyloid) or tangles with this latter antibody may represent nonspecific cross-reactivity, an interpretation consistent with findings reported by Wilhelmus and colleagues [27], who likewise failed demonstrate any immunostaining of these structures with HSP27. Our presently reported observations that no HSP27 immunostaining was seen in Pick bodies in patients with FTLD-tau, or in TDP-43 immunoreactive neuronal cytoplasmic inclusions in patients with FTLD-TDP or MND, would support this conclusion. Interestingly, in previous studies where HSP27 immunostaining of astrocytes has been reported [12,27], this has taken the form of a homogeneous, diffuse cytoplasmic staining rather than one with pronounced granularity as demonstrated here. Again, different antibody specificities may be responsible, picking up HSP27 proteins either in a different conformational state or in aggregated forms.

The pattern of HSP27 immunoreactivity in cells may reflect an activation of protein quality control systems consequent upon damage to neurons or a response to the presence of accumulated tau/TDP-43 proteins. It is increasingly clear that the cooperation of proteolytic machineries, such as the proteasome and lysosome, and chaperone systems is required to prevent the aggregation of potentially toxic misfolded proteins. If such misfolded proteins escape appropriate refolding by chaperone systems, the proteasome and lysosome systems will be recruited to degrade them. However, if the production of such misfolded proteins overwhelms the capacity of the protein quality control systems, protein aggregates will form.

Within our control group, there was variability in the expression of HSP27. In some control subjects similar (to AD and FTLD), although less intense, HSP27 changes were present in the cytoplasm of pyramidal neurons of the cerebral cortex, and again as diffuse clusters of similar granules within glial cells. Such changes probably reflect the mild AD-type pathological changes present in such control subjects. In a recent study of patients with amnestic mild cognitive impairment (aMCI), a transitional stage between normal ageing and AD, there was a significant increase (200%) in the expression of HSP27 in the hippocampus in the aMCI group compared to controls [28], which would be consistent with these ‘intermediate’ histological findings.

It is presently unclear which cellular compartment or substructure might be associated with HSP27 immunostaining. The granularity of the staining suggests a phagocytotic location. Consequently, we stained sections in which there was an abundance of HSP27-immunoreactive granules with LAMP1 (a lysosomal marker) and LCA3 (a marker of autophagy) antibodies. LAMP1 detected granular structures in neurons and glia similar to those detected by HSP27, but none such were demonstrated in LCA3 immunostaining. These observations suggest that HSP27 immunoreaction is possibly associated with the cells’ lysosomal compartments and may represent accumulated protein undergoing or awaiting degradation or the product of a failed degradation process. Nonetheless, a proteasomal location is also possible, and the pattern of immunoreactivity could represent proteasomes clogged or choked with accumulated protein. However, the fact that the ‘granules’ were not immunostained with either p62 or ubiquitin antibodies suggests that the HSP27 immunolabelled material does not contain either of these proteins, which act as molecular ‘flags’ for proteasomal degradation. Therefore, the subcellular localisation of HSP27 immunostaining, and its role in the neurodegenerative cascade, remains uncertain though may possibly be lysosomal.

## Conclusions

In this study, we show that there are distinct patterns of HSP27 immunostaining with respect to different types of neurons or glial cells in different brain regions in patients with neurodegenerative disease. In general, patients with AD and FTLD were more severely affected than were patients with MND and control subjects; however, there was no association with FTLD histological subtype. Moreover, the ‘classic’ neuropathological changes in FTLD, AD and MND were not immunoreactive to HSP27, with the lack of HSP27 immunostaining of ‘classic’ pathology suggesting that HSP27 does not play a direct role in the formation of these structures. Therefore, HSP27 immunostaining in neurodegenerative disease likely reflects the extent of ongoing neurodegeneration in affected brain areas and is not specific to FTLD, AD or MND. It may represent an accumulation of misfolded, damaged or unwanted proteins, waiting for degradation via proteolytic mechanisms, though the precise pathway that this might enter, or fail to enter, for such a purpose remains unclear.

## Abbreviations

AD: Alzheimer’s disease
Aβ: Amyloid-β protein
FTLD: Frontotemporal lobar degeneration
HSP: Heat shock protein
MND: Motor neuron disease
PD: Parkinson’s disease
TDP-43: Transactive response DNA binding protein 43
